# Impact of COVID-19 on diagnosis of tuberculosis and tuberculosis infection in South America, Asia, and Africa

**DOI:** 10.36416/1806-3756/e20240077

**Published:** 2024-06-21

**Authors:** Denise Rossato Silva, Rosella Centis, Lia D’Ambrosio, Fernanda Carvalho de Queiroz Mello, Giovana Rodrigues Pereira, Sarita Aguirre, Seif Al-Abri, Khalsa Al-Thohli, Fatma Al Yaquobi, Marianne Calnan, Rosarito Coronel Teixeira, Sandra Inwentarz, Domingo Juan Palmero, Alberto Piubello, Samridhi Sharma, Mahamadou Bassirou Souleymane, Alphazazi Soumana, Sai Meng Tham, Pei Min Thong, Zarir F Udwadia, Martin van den Boom, Giovanni Sotgiu, Giovanni Battista Migliori

**Affiliations:** 1. Faculdade de Medicina, Universidade Federal do Rio Grande do Sul - UFRGS - Porto Alegre (RS) Brasil.; 2. Servizio di Epidemiologia Clinica delle Malattie Respiratorie, Istituti Clinici Scientifici Maugeri - IRCCS - Tradate, Italia.; 3. Public Health Consulting Group, Lugano, Switzerland.; 4. Instituto de Doenças do Tórax - IDT - Faculdade de Medicina, Universidade Federal do Rio de Janeiro - UFRJ - Rio de Janeiro (RJ) Brasil.; 5. Ambulatório de Tuberculose de Alvorada, Alvorada (RS) Brasil.; 6. Ministerio de Salud Pública y Bienestar Social, Asunción, Paraguay.; 7. Royal Hospital, Ministry of Health, Muscat, Sultanate of Oman.; 8. TB and Acute Respiratory Diseases Section, Department of Communicable Diseases, Directorate General of Disease Surveillance and Control, Ministry of Health, Sultanate of Oman.; 9. University Research Co. LLC, Manila, Philippines.; 10. National Institute of Respiratory Diseases and the Environment - INERAM - Asunción, Paraguay.; 11. Instituto Vaccarezza - UBA - Buenos Aires, Argentina.; 12. Damien Foundation, Brussels, Belgium.; 13. P.D. Hinduja National Hospital and Medical Research Centre, Mumbai, India.; 14. Damien Foundation, Niamey, Niger.; 15. National Tuberculosis Programme, Niamey, Niger.; 16. Division of Infectious Diseases, Department of Medicine, National University Hospital, Singapore.; 17. Department of Medicine, Infectious Disease Translational Research Programme, National University of Singapore, Yong Loo Lin School of Medicine, Singapore.; 18. World Health Organization, Regional Office for the Eastern Mediterranean Region, Cairo, Egypt.; 19. Clinical Epidemiology and Medical Statistics Unit, Department of Medical, Surgical and Experimental Sciences, University of Sassari, Sassari, Italy.

## TO THE EDITOR:

During the COVID-19 pandemic, most countries implemented public health measures intended to contain the spread of SARS-CoV-2, such as travel restrictions, stay-at-home measures, use of face masks, among others.^1^ Because of the pandemic, several disruptions occurred in healthcare services. tuberculosis-related services were severely affected, with closing of health care units or limited patient access, reduction of the number of healthcare professionals due to relocation to COVID-19 care, and decreased healthcare demand by patients due to fear of exposure to SARS-CoV-2, with a consequent decrease in diagnosed cases due to diagnostic delay.^1^ ^-^ ^4^

The WHO reported that tuberculosis notifications dropped 18% from 2019 to 2020 (from 7.1 to 5.8 million cases), with a partial recovery in 2021, but still without reaching pre-pandemic values.^5^ A global study,^2^ coordinated by the Global Tuberculosis Network and involving 43 centers from 19 countries, demonstrated that newly diagnosed tuberculosis disease, drug-resistant tuberculosis (DR-TB), tuberculosis deaths, outpatient clinic visits, and newly diagnosed tuberculosis infection were reduced.

Information on the effects of COVID-19 on tuberculosis, specifically in South America, Asia, and Africa, is very limited. Therefore, the aim of this study was to compare the prevalence of tuberculosis disease (new or retreatment cases), newly diagnosed tuberculosis infection, and DR-TB between the years 2020 and 2019 in eight countries located in South America (Argentina, Brazil, Paraguay), Asia (India, Oman, the Philippines, Singapore), and Africa (Niger).

Data previously collected by the Global Tuberculosis Network were used.^2^ The following variables were collected monthly: total number of tuberculosis cases, including new diagnoses and recurrences; number of DR-TB; and number of tuberculosis deaths. The coordinating and the participating centers had ethics clearance in accordance with their institutional regulations. Data were collected from January 01, 2019, to December 31, 2020. Statistical analysis was performed using IBM SPSS Statistics, version 22.0 (IBM Corporation, Armonk, NY, USA). Data were presented as medians and interquartile ranges. Variables were compared using the Wilcoxon test. A two-sided p < 0.05 was considered significant.

**Figure 1:**
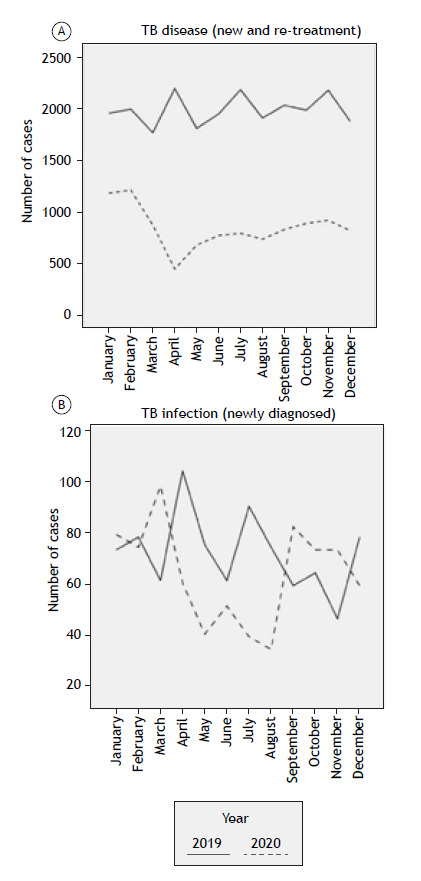
Number of tuberculosis (TB) disease cases (new and retreatment; in A) and of TB infection cases (in B) in 2019 and 2020, by month, in eight South American, Asian, and African countries.

The median number of tuberculosis disease cases decreased in 2020 (33.0 [19.3-105.8] vs. 41.0 [25.0-114.0], p = 0.029). Newly diagnosed tuberculosis infections did not significantly decrease from 2019 to 2020 (7.0 [3.3-28.0] vs. 6.0 [2.0-23.0]; p = 0.470). No differences were found in the number of DR-TB cases and tuberculosis deaths (p = 0.293 and p = 0.433, respectively). In India, the number of DR-TB cases was significantly lower in 2020 (86.0 [60.3-106.3] vs. 323.5 [308.3-354.5]; p < 0.0001). Tuberculosis deaths in Paraguay decreased in 2020 (16.5 [12.5-19.8] vs. 26.5 [22.3-27.0]; p < 0.0001; Figure 1).

Our findings are similar to those described in previous studies.^6^ ^,^ ^7^ demonstrating a reduced number of tuberculosis notifications during the first year of the COVID-19 pandemic. Morena et al.^7^ evaluated the impact of the COVID-19 pandemic on pulmonary tuberculosis using artificial intelligence in the region of Castilla-La Mancha in Spain: a significant decrease in the incidence of pulmonary tuberculosis at the start of the COVID-19 outbreak was found. In another study,^6^ changes of reported tuberculosis incidence and mortality before and during the pandemic were described in China from January of 2015 to January of 2023. In the present study, we found no differences in the number of tuberculosis deaths when comparing 2019 with 2020, except in Paraguay where the number of deaths decreased in 2020.

In summary, a decrease in the number of tuberculosis disease cases notified in South America, Asia, and Africa was proven in 2020 if compared with that notified in 2019. Tuberculosis infection decreased from 2019 to 2020 without any statistical significance. Further monitoring will be necessary, as an increase of several indicators of tuberculosis disease and infection may be expected in future years.
